# Soluble Insulin Receptor Levels in Plasma, Exosomes, and Urine and Its Association With HIV-Associated Neurocognitive Disorders

**DOI:** 10.3389/fneur.2022.809956

**Published:** 2022-06-02

**Authors:** Yisel M. Cantres-Rosario, Valerie Wojna, Rafael Ruiz, Bexaida Diaz, Miriam Matos, Rosa J. Rodriguez-Benitez, Elaine Rodriguez, Richard L. Skolasky, Yamil Gerena

**Affiliations:** ^1^NeuroHIV Research Program, School of Medicine, University of Puerto Rico, San Juan, PR, United States; ^2^Division of Neurology, Internal Medicine Department and NeuroHIV Research Program, School of Medicine, University of Puerto Rico, San Juan, PR, United States; ^3^Department of General Social Sciences, University of Puerto Rico, San Juan, PR, United States; ^4^Orthopaedic Surgery and Physical Medicine & Rehabilitation, Johns Hopkins University, Baltimore, MD, United States; ^5^Department of Pharmacology and Toxicology, School of Medicine, NeuroHIV Research Program, Pharmacology Department, University of Puerto Rico, San Juan, PR, United States

**Keywords:** insulin receptor, exosome, urine, plasma, cognitive dysfunction, biomarker, women

## Abstract

**Background:**

HIV-associated neurocognitive disorders (HAND) are one of the HIV-associated comorbidities affecting 20–50% of the people with HIV (PWH) infection. We found that the soluble insulin receptor (sIR) levels in plasma and cerebrospinal fluid (CSF) were significantly higher in HIV-infected women. The mechanism of sIR release into the plasma remains unknown, but the detection of the sIR in exosomes may uncover novel mechanisms of sIR secretion from HIV-infected cells and its contribution to HIV disease progression and HAND development. Quantification of sIR in urine may represent a less invasive and more accessible diagnostic tool. Our objective was to quantify sIR levels in plasma, plasma-derived exosomes, and urine, and evaluate their association with HAND and renal function.

**Methods:**

We measured full-length sIR in the plasma and urine of 38 controls and 76 HIV-infected women by ELISA, and sIR, HIV-1 Tat, and reactive oxygen species (ROS) in exosomes by flow cytometry.

**Results:**

Plasma and exosomes with sIR were significantly higher in HIV-infected women when compared with controls and HAND. Exosomal sIR positively correlated with exosomal ROS and exosomal HIV-1 Tat in HIV-infected women. Exosomal ROS was significantly higher in HIV-infected women with more symptomatic cognitive impairment. Plasma-derived exosomes exhibited significantly higher levels of astrocyte (GFAP) and neuronal (L1CAM) markers in HIV-infected women, confirming the presence of circulating CNS-derived exosomes in the blood of HIV-infected women. Urine sIR positively correlated with eGFR in controls, but not in HIV-infected women, regardless there was no significant difference in renal function as determined by the estimated glomerular filtration rate (eGFR, *p* = 0.762). In HIV-infected women, higher plasma sIR correlated with lower urine sIR that could suggest sIR retention in blood or decreased renal filtration.

**Discussion:**

Higher plasma sIR levels and their correlation with ROS in plasma-derived exosomes with HAND suggest a combined role of metabolic disturbances, oxidative stress, exosome release, and cognitive decline. Communication between CNS and periphery is compromised in PWH, thus plasma-derived exosomes may shed light on disrupted cellular mechanisms in the brain of PWH. High plasma and low urine sIR levels could suggest sIR retention in blood or decreased renal filtration.

## Introduction

The introduction of combined antiretroviral therapy (cART) to HIV treatment has improved the life quality and expectancy of people with HIV infection (PWH), transforming the disease into a chronic condition. However, this increased life expectancy combined with the long-term infection and cART, has increased the risk of comorbidities such as cardiovascular diseases, chronic inflammation, changes in body composition and metabolic activity, and insulin resistance (1–4). In the cART era, no clear evidence exists to understand the cellular and molecular basis of these comorbid conditions. Thus, there is a need to identify the factors that contribute to the disruption in insulin signaling and metabolic pathways and determine how these contribute to cognitive impairment in PWH. Furthermore, there are no biomarkers that could be used in routine clinical diagnosis of HAND, and the ones that have been identified show little correlation with the disease (5–11). There has been an urge to identify a biomarker since early diagnosis and interventions may help prevent the progression from mild to more severe forms of HAND or improve HAND (12).

Previously, we demonstrated that HIV-infected women with cognitive impairment have higher levels of soluble insulin receptor (sIR) in plasma and cerebrospinal fluid (CSF), when compared to HIV-negative women, as well as lower levels of insulin receptor substrate 1 (IRS-1) tyrosine phosphorylation in plasma (13–15). Metabolic enzymes are also altered in patients with HAND (16, 17). The mechanisms responsible for the secretion of the full-length sIR to the plasma of HIV-infected women with HAND remain unknown. The exosome-mediated release is one of the mechanisms responsible for the secretion of soluble full-length receptors and viral proteins from infected cells (18–20). These extracellular vesicles are single membrane organelles (~40–100 nm) produced by healthy and virus-infected cells and play different roles in normal and pathophysiological conditions (21, 22). They contain lipids, proteins, and nucleic acids (mRNAs and miRNAs). Some of these exosomal components have been explored as potential biomarkers to monitor cellular disease states. Extracellular vesicles have also been implicated in the processing of proteins associated with neurological diseases (18, 21, 23–25). Once the exosomes are released, they can exert regulatory influences on target cells such as the modulation of host immune responses and microbial pathogenesis (26). Their secretion from the HIV-infected cells may have different cargo and functions than those released from uninfected cells. In fact, HIV-1 infected monocyte-derived macrophages (MDM) release a higher number of exosomes compared to non-infected cells (20). In this study, we quantified sIR in exosomes derived from plasma obtained from HIV-infected women on cART and investigated if sIR levels were associated with the presence and severity of HAND. Since HIV infection can trigger oxidative stress (27), we measured the levels of reactive oxygen species (ROS) inside exosomes as well as the presence of the HIV-1 Tat protein. In addition, we measured astrocyte and neuronal markers in these plasma exosomes to determine their origin.

Although plasma, CSF, and exosomal sIR could serve as a biomarker for the presence and severity of HAND, there is a need for more accessible and less invasive diagnosis methods. Measuring full-length sIR in urine can reduce not only the risk of infection and pain in a blood collection method but also the sampling cost. For these reasons, in this study, we also investigated if sIR was present in the urine of HIV-infected women and their association with HAND and renal function. The quantification of sIR subunits in the urine has been previously explored as a good marker for evaluating diabetes risk (28, 29). It is likely that our findings will provide evidence to investigate other pathophysiological mechanisms involved in the secretion of sIR and viral proteins in these patients. This receptor may contribute to the cognitive decline observed in this population (13–15). The identification of novel mechanisms will serve to design innovative approaches and treatment regimens for the prevention and treatment of insulin resistance and cognitive impairment in HIV-infected patients.

## Materials and Methods

### Participants and Study Design

This was a cross-sectional study using patient-database information and the sample repository of the Hispanic-Latino Longitudinal Cohort of Women. This study was approved by the University of Puerto Rico Medical Sciences Campus (UPR-MSC) Institutional Review Board (#1330315), and all the participants signed written informed consent. This is a unique cohort of Hispanic HIV-infected women characterized by viral and immune profiles, neurological exams, and neuropsychological tests. A total of eighty-six (*N* = 86) HIV-infected women without a history of diabetes and thirty-five (*N* = 35) HIV-negative controls were evaluated, as previously described (13, 30). The HIV-infected women were grouped according to their cognitive performance as having normal cognition (N; *n* = 44), asymptomatic impairment (ANI; *n* = 10), and symptomatic impairment (SI, mild neurocognitive disorder [MND, *n* = 18] plus HIV-associated dementia [HAD, *n* = 14]; *n* = 32). The estimated glomerular filtration rate (eGFR) was calculated from serum creatinine (mg/dL) using a validated Chronic Kidney Disease Epidemiology Collaboration equation validated in 2009 by Levey et al. in a population of Caucasian and African–American study participants (31).

### Characteristics of HIV-Infected Women

When the HIV-infected women were grouped by cognitive performance, no significant differences were observed between groups regarding age, body mass index (BMI), current CD4 cell count, Homeostatic Model Assessment for Insulin Resistance (HOMA-IR), plasma viral load, blood urea nitrogen (BUN), creatinine, estimated glomerular filtration rate (eGFR), or toxicology (*p* > 0.05). No proteinuria or albuminuria was observed among the participants. Most HIV-infected women were using antiretroviral therapy (ART) (89.5%, or 77 of the 86 women) with protease inhibitors (51%, or 39 of the 77 women taking ART). There was no significant difference between groups of HAND severity in either use of ART, protease inhibitor, or CNS penetration effect [CPE, (32)] (*p* > 0.05; Table 1). However, twenty (*n* = 20) of the HIV-infected patients were coinfected with hepatitis C virus (HCV). HIV viral load was detectable in thirty-two (*n* = 32) women, which accounts for 37.2% of the HIV-infected subjects in this study.

**Table 1:** Characteristics of HIV-infected women.

	**HIV-negative controls**	**Normal cognition**	**Asymptomatic**	**Symptomatic**	***p*-value**
	***N =* 35**	***N =* 44**	***N =* 10**	***N =* 32**	
Age (yrs.)	45.5 (31.8, 52.3)	46.5 (39.0, 51.0)	44.0 (37.3, 52.0)	42.0 (35.3, 48.5)	0.682
Total CD4 (cells/mm^3^)	N/A	688 (340, 938)	428 (225, 861)	617 (467, 920)	0.308
Plasma HIV RNA (Log copies/mL)	N/A	1.3 (1.3, 2.4)	1.3 (1.3, 3.6)	1.6 (1.3, 4.5)	0.453
Hepatitis C virus positive (%)	0 (0%)	9 (20.5%)	3 (30.0%)	8 (25.0%)	0.101
Body mass index	29.7 (25.9; 34.5)	30.7 (24.2, 36.6)	26.7 (22.8, 31.5)	29.8 (24.2, 38.1)	0.714
Toxicology Positive (%) Marihuana (n) Cocaine (n) Both (n) Unspecified (n)	0 (0%) 0 0 0 0	10 (22.7%) 1 5 2 2	2 (20.0%) 0 2 0 0	8 (25.0%) 2 3 1 1	0.050
Antiretroviral therapy treated (%)	N/A	38 (86.4%)	9 (90%)	30 (93.8%)	0.586
Protease inhibitors included in cART regimen (%)	N/A	19 (43.2%)	5 (50.0%)	15 (46.9%)	0.906
CNS penetration effect score	N/A	7 (7, 8)	7 (6.5, 7.0)	7 (6.8, 7.3)	0.399
eGFR (mL/min/1.73 m^2^)	88.4 (84.8, 92.3)	94.3 (84.9, 126.6)	81.7 (69.3, 130.0)	94.2 (75.8, 123.6)	0.762
Creatinine (mg/dL)	0.7 (0.6, 0.7)	0.7 (0.6, 0.8)	0.8 (0.6, 0.9)	0.7 (0.6, 0.8)	0.528
BUN (mg/dL)	12.0 (10.0, 16.0)	12.0 (9.0, 15.0)	14.5 (12.0, 17.0)	13.0 (10.0, 15.0)	0.222
Plasma sIR (MFI)	10,109 (9,586, 10,341)	10,515 (10,073,10,856)	10,704 (9,786, 11,225)	10,533 (10,084, 10,762)	** 0.001
Urine sIR (MFI)	9,192 (8,711, 9,752)	9,117 (8,679, 9,795)	9,771 (8,948, 10,411)	9,350 (9,223, 9,635)	0.171
HOMA-IR	1.7 (1.2, 3.2)	2.0 (1.4, 3.0)	1.5 (1.4, 2.3)	1.6 (1.0, 2.5)	0.578

Median (25th, 75th percentile), **Kruskal-Wallis test p-value.

### Blood and Urine Sample Preparation

Fresh blood samples were drawn from each patient and collected in acid citrate dextrose (ACD) tubes. Plasma samples were obtained after centrifuging the blood samples twice at room temperature for 10 min at 355 × g. A plasma aliquot of 700 μl was used for viral load determination, aliquots of 500 μl were used to determine sIR full-length levels, and aliquots of 1 ml were used to determine sIR full-length levels in exosomes. Urine samples (10 ml) were collected from each patient, centrifuged at 2,000 × g for 10 min to remove debris, and analyzed for sIR levels as described below.

### Soluble Insulin Receptor Full-Length (sIR-αβ, Intact) Assay

Plasma and urine sIR full-length levels were determined as previously published (13). Briefly, the levels were determined by sandwich ELISA using anti-IR antibodies (Abcam, Cambridge, MA) and a FITC-secondary antibody (Abcam, Cambridge, MA). The samples were analyzed in a Cytofluor 4000 (Applied Biosystems, CA) using 485/530 nm excitation/emission filters.

### Determination of sIR, HIV-1 Tat, ROS, GFAP, and L1CAM Levels in Exosomes

Exosomes were isolated from plasma by ultracentrifugation (100,000 x g) and then incubated with aldehyde/sulfate beads 4%w/v (1.4 × 10^9^/ml beads; Life Technologies) overnight at 4**°**C (24, 33, 34). Exosome-coated beads (4 μg/μl) were incubated with CD63-Alexa 647 and Rab-5b- PE antibodies (Abcam, MA) for 1 h at 4°C (35). Then, the exosomes were permeabilized using the BD Cytofix Cytoperm Kit (BD biosciences, CA) for 20 min at 4°C and incubated with corresponding primary and secondary antibodies. For insulin receptors, exosomes were incubated with anti-IR β-subunit antibody (Abcam, MA), followed by a FITC-conjugated anti-mouse secondary antibody as previously (13). For ROS, exosomes were stained using the Oxidative Stress Detection Reagent (Green Fluorescent) as described by the manufacturer (ENZO Life Sciences, Farmingdale, NY). For HIV-1 Tat protein, exosomes were also permeabilized as described above and labeled using anti-HIV-1 Tat- FITC fluorescent antibody (Abcam, MA). Glial fibrillary acidic protein (GFAP) is an astrocyte marker, which was detected using Recombinant Alexa Fluor^®^ 488 anti-GFAP rabbit monoclonal antibody, EPR1034Y clone (Abcam, MA). L1 cell adhesion molecule protein (L1CAM) is a protein associated with neurons, which was detected using PE-conjugated anti-L1CAM mouse monoclonal antibody, clone 5G3 (Abcam, MA). Anti-GFAP and anti-L1CAM both were used at dilutions of 5 μl per 10^6^ cells/ml suspensions. The samples were analyzed using a FACSAria flow cytometer (BD Biosciences, CA). Two measurements were performed in the exosomes, sIR, HIV-1 Tat, and ROS levels per exosome and the percentage of exosomes with sIR, HIV-1 Tat, and ROS.

### Plasma CD163 ELISA

CD163 was measured in plasma samples collected in ACD blood collection tubes using solid-phase sandwich ELISA assay (R&D systems Human CD163 Quantikine ELISA Kit #DC1630) following the manufacturer's instructions. The minimum sample dilution was 1:10, and the maximum was 1:40. Data were reported as mean (ng/ml) and standard deviation.

### Flow Cytometry

Flow cytometric analyses were carried out using a FACSCelesta cytometer (BD Biosciences, CA). Fluorescent exosomes labeled with Anti-CD63-Alexa647 (FL4 channel, 661/16 nm), Anti-Rab5b-PE (FL2 channel, 585 nm), and Anti-IRβ-FITC, Anti-Tat-FITC or ROS fluorescent reagent (FL1 channel, 525 nm) were identified first in the FL2 vs. FL4 dot plots, and then the levels of sIR, HIV-1 Tat, and ROS in the exosomes were determined from the median peak channel of the FL1-histograms. The FACSDIVA software (BD Biosciences, CA) was used for data acquisition and multivariate analysis. Cells were gated in forward/side scatter dot plots. Data on scatter parameters and histograms were acquired in log mode. A total of fifty thousand events were evaluated for each sample and the median peak channel obtained from the histograms was used to determine the levels of sIR in exosomes.

### Statistical Analyses

Age, CD4 count, viral load, BMI, toxicology, cART treatment, and use of protease inhibitor in cART regimen (yes or no), Homeostatic Model Assessment for Insulin Resistance (HOMA-IR), and CNS penetrance effectiveness (CPE) score variables were compared among the three HIV-infected groups stratified by cognitive impairment as having normal cognition (N), asymptomatic impairment (ANI), or symptomatic impairment (SI), and reported as the median and interquartile range (Table 1). D'Agostino and Pearson omnibus normality test was performed on all variables and the outcomes were measured. Because the clinical data and parameters measured in the samples were not always normally distributed, comparisons were made using the Mann–Whitney tests for two samples and Kruskal–Wallis with Dunn's multiple comparisons test for more than two sample groups. Correlations were performed using Spearman's rank test. All statistical analyses were performed with IBM SPSS Statistics v24.0 and GraphPad Prism v6.0 softwares. Statistical significance was considered at *p* < 0.05.

## Results

### Full-Length sIR Levels Were Increased in Plasma of HIV-Infected Women

**Figure 1:**
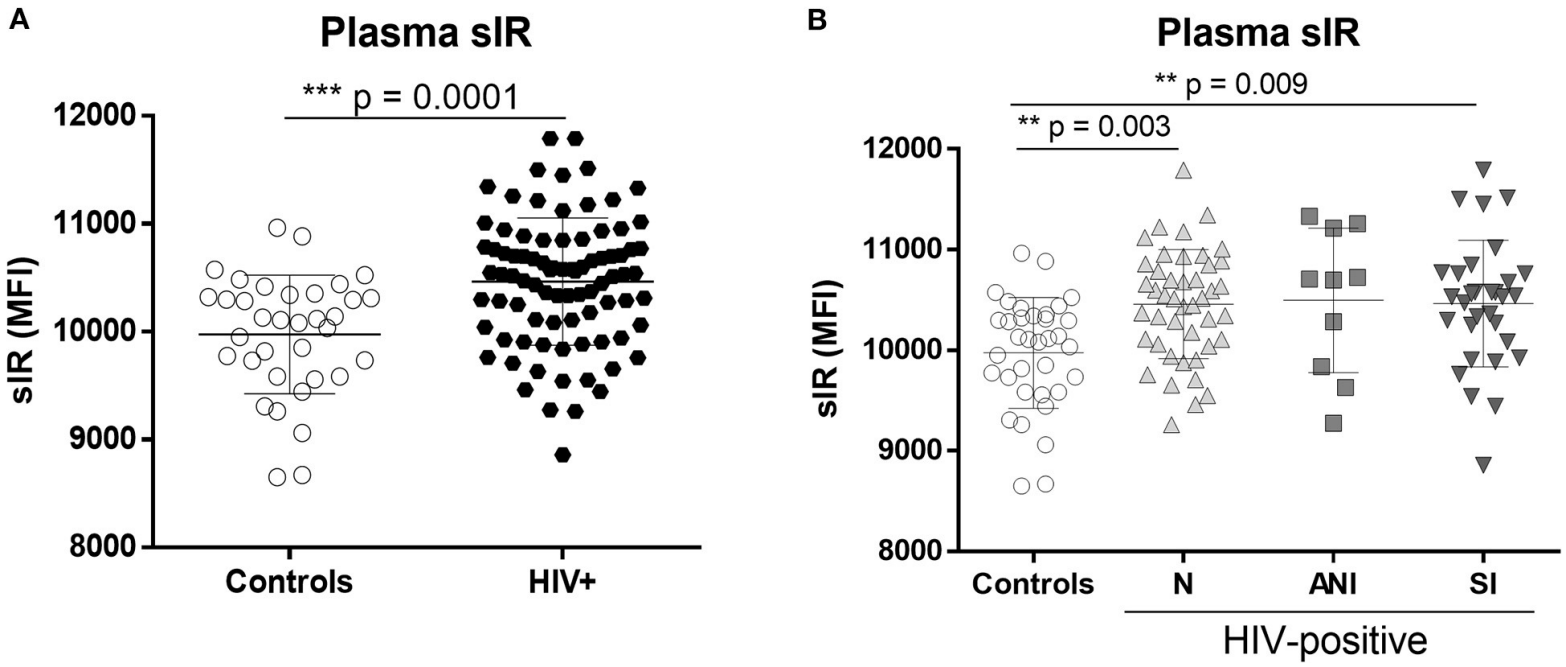
Levels of the soluble insulin receptor in plasma of control and HIV-infected women. sIR was measured in the plasma of HIV-negative (controls *n* = 35) and HIV-infected (*n* = 86) women by flow cytometry **(A)**. HIV-infected women were stratified by HAND status as normal cognition (N) (*n* = 44), asymptomatic cognitive impaired (ANI) (*n* = 10), and symptomatic impaired (SI; MND and HAD) (*n* = 32) **(B)**. Graphs show mean and standard deviation [Controls (9,975 ± 549.2), N (10,459 ± 541.6), ANI (10,497 ± 718.7) and SI (10,464 ± 630.8)] results were graphed as mean fluorescence intensity (MFI) units. Analysis was performed using the Unpaired *t*-test and Kruskal–Wallis test with Dunn's multiple comparisons.

As previously published (13–15), full-length sIR levels in the plasma of HIV-infected women were significantly increased relative to controls (*p* < 0.001, Figure 1A), with significant differences among the control group and HIV-infected women stratified by cognitive impairment (C vs. N, *p* = 0.003, C vs. SI, *p* = 0.009) (Figure 1B). No differences in plasma sIR were observed among HIV patients.

### Exosomes With sIR Were Detected in HIV-Infected Women and Associated With HAND

**Figure 2:**
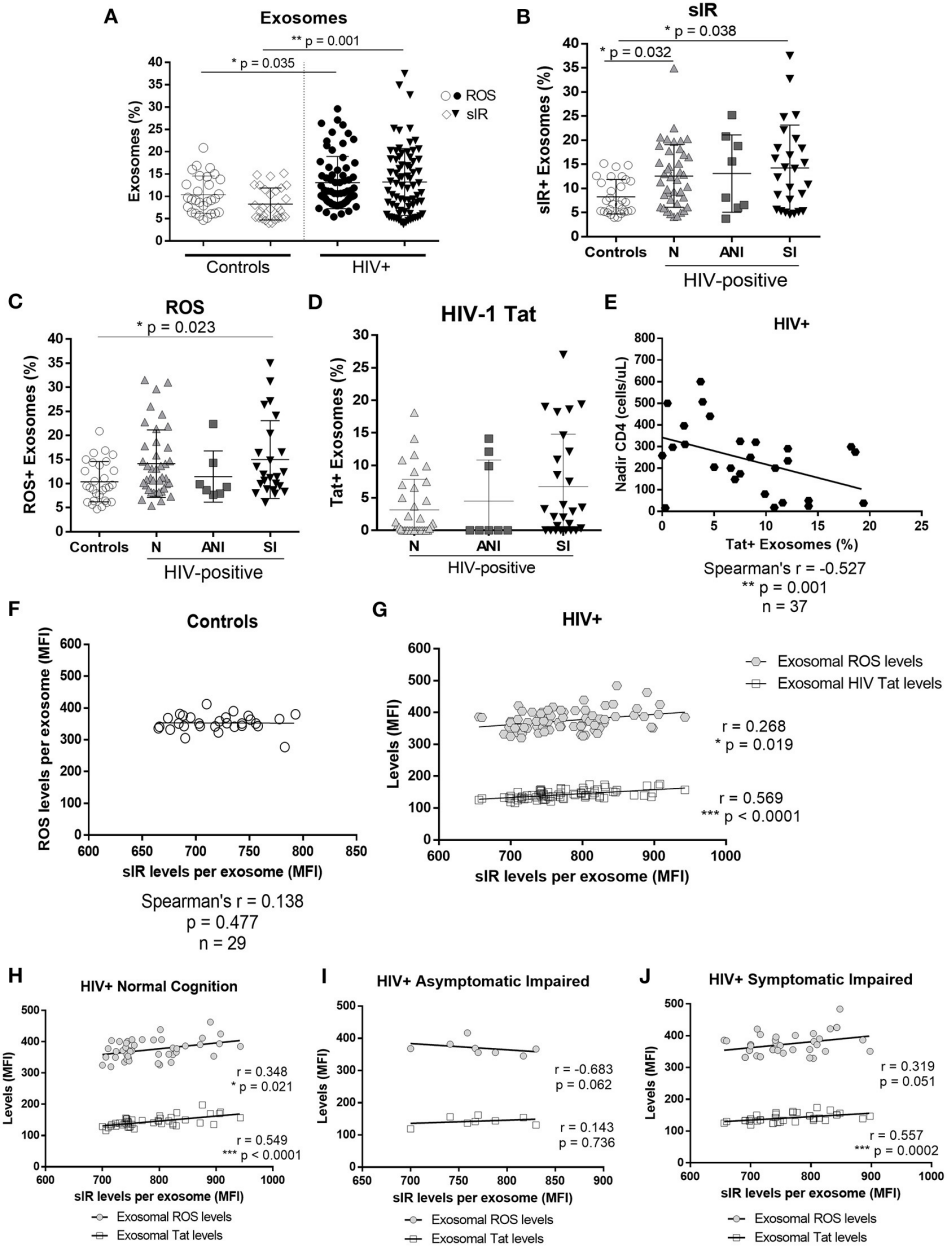
Exosomal sIR, HIV-1 Tat, and ROS in control and HIV-infected women. Levels of sIR+, HIV-1 Tat+, and ROS+ exosomes (exosome %) as well as the levels inside the exosomes were measured in exosomes isolated from the plasma of HIV-negative controls and HIV-infected women by flow cytometry **(A)**. HIV-infected women were stratified by HAND status as normal cognition (N), asymptomatic cognitive-impaired (ANI), and symptomatic impaired (SI; MND and HAD) **(B–D)**. Flow cytometry results were graphed as mean fluorescence intensity (MFI). Graphs show the mean and standard deviation. For percentage of sIR+ exosomes **(B)**: [Controls (8.29 ± 3.58), N (12.59 ± 6.48), ANI (13.11 ± 8.01), and SI (14.25 ± 8.89)]; for ROS+ exosomes **(C)**: [Controls (10.40 ± 4.18), N (14.15 ± 7.00), ANI (11.46 ± 5.31), and SI (15.01 ± 8.08)]; and for HIV-1 Tat+ exosomes **(D)**: [N (3.16 ± 4.74), ANI (4.51 ± 6.33), and SI (6.75 ± 8.06)]. Kruskal–Wallis and Dunn's multiple comparisons were used. Spearman's correlation was also conducted between HIV-1 Tat+ exosomes (%) and nadir CD4+ levels in HIV-infected women in samples where HIV-1 Tat+ exosomes were detectable **(E)**. Spearman's correlations between exosomal levels of sIR, ROS, and HIV-1Tat (HIV-infected samples only) were conducted in controls **(F)** and HIV-infected exosomes **(G)**. Stratified by cognitive performance, correlations of exosomal sIR with exosomal ROS and HIV-1 Tat were determined in HIV-infected women with normal cognition **(H)**, asymptomatic impairment **(I)**, and symptomatic impairment **(J)** using a Spearman's correlation.

HIV-infected women had significantly higher levels of exosomes with sIR than did controls (*p* = 0.001) as well as exosomes containing ROS (*p* = 0.035; Figure 2A). We observed a significant association between exosomes with sIR and HIV-1 infection, where N and SI showed a higher percentage of exosomes with sIR when compared with controls (N vs. C, *p* = 0.032 and SI vs. C, *p* = 0.038, Figure 2B). The SI group presented higher levels of ROS containing exosomes than controls (SI vs. C, *p* = 0.023, Figure 2C), but no significant differences were observed among the HAND groups. No significant differences were observed in the percentage of exosomes containing HIV-1 Tat between the HAND groups (Figure 2D). However, in HIV-infected patients, a higher percentage of HIV-1 Tat+ exosomes correlated with lower levels of nadir CD4+ T cells (Figure 2E, *p* < 0.001). The levels of ROS and sIR inside exosomes isolated from controls showed no correlation (Figure 2F; *p* = 0.477). However, in HIV-infected women, the levels of sIR inside exosomes correlated with exosomal ROS (*p* = 0.019) and HIV-1 Tat levels (*p* < 0.0001; Figure 2G). When stratified by normal cognition and cognitive impairment, the correlation between sIR and ROS inside exosomes was maintained among normal cognitive HIV-infected women (Figure 2H; *p* = 0.021) but not in cognitive-impaired women (Figures 2I,J). The infected correlation between sIR and HIV-1 Tat was significant in both normal cognitive (Figure 2H) and cognitive-impaired women (Figures 2I,J; *p* < 0.0001).

### Plasma sIR Levels and Exosomal sIR Levels Were Not Associated

**Figure 3:**
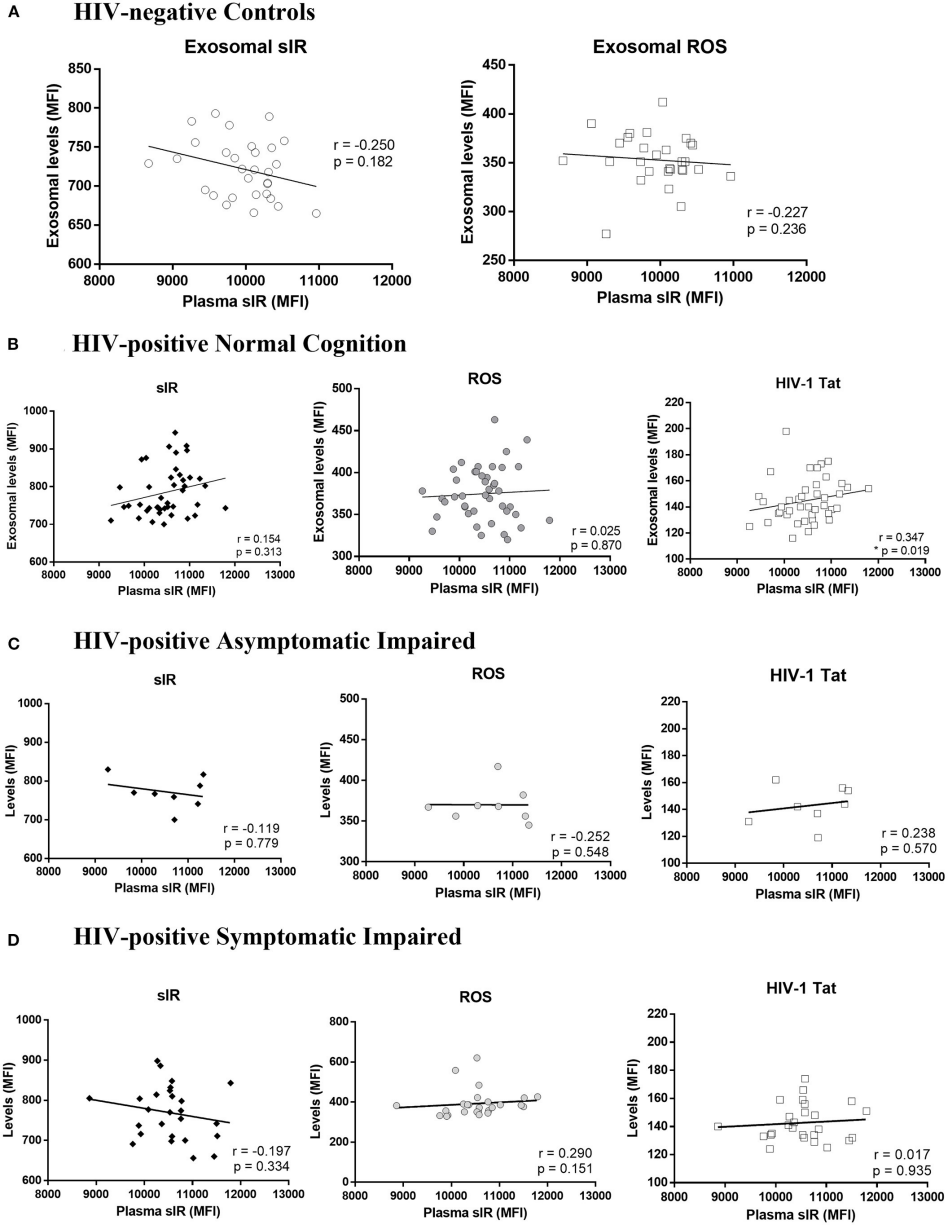
Association between soluble insulin receptor levels in exosomes and plasma. Associations between plasma sIR and exosomal levels of sIR, ROS, and HIV-1 Tat (levels per exosome) were determined by Spearman's correlations tests in HIV-negative controls **(A)** and HIV-infected women with normal cognition **(B)**, asymptomatic impairment **(C)**, and symptomatic impairment **(D)**.

Since the exosomes were purified from the plasma, we performed Spearman's correlation tests to determine if the levels of sIR in exosomes are associated with levels of sIR in plasma. There was no correlation between plasma and exosomal sIR in controls or HIV-infected women stratified by HAND (*p* >0.05; Figures 3A–D). HIV-1 Tat and ROS levels inside exosomes did not correlate with plasma sIR in controls nor HIV-infected women. Among the HIV-infected women with normal cognition, the levels of sIR in plasma positively correlated with the levels of HIV-1 Tat (Figure 3B; *p* = 0.019). This was not observed in those with cognitive impairment (Figures 3C,D).

### Astrocyte and Neuron-Derived Exosomes Are Increased in the Plasma of HIV-Infected Patients

**Figure 4:**
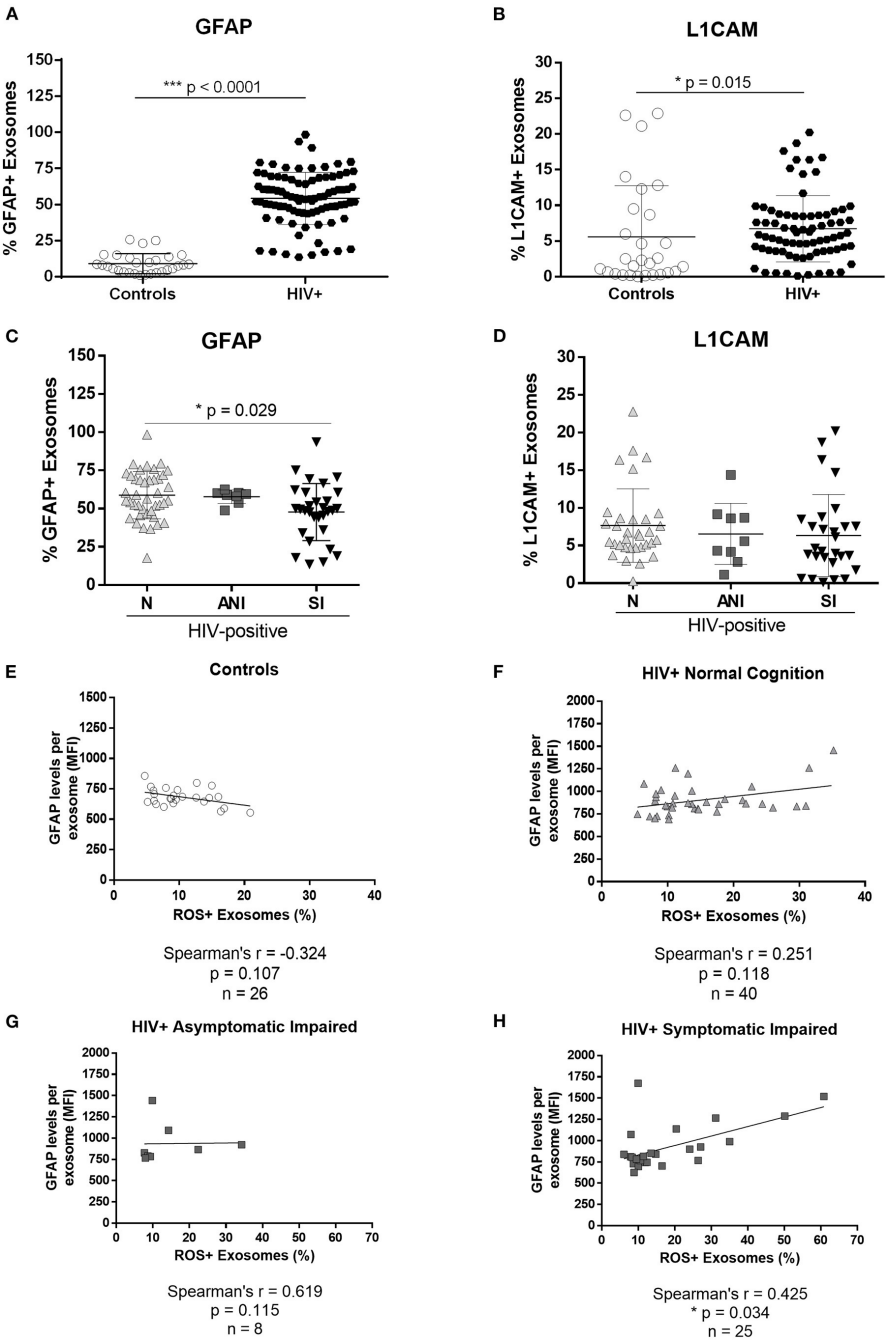
Astrocyte and neuronal markers in plasma-derived exosomes from HIV-infected women. Glial fibrillary acidic protein (GFAP), an astrocyte marker, and L1 cell adhesion molecule protein (L1CAM), a neuronal marker, were measured in plasma-derived exosomes by flow cytometry. Mann–Whitney tests were used to compare GFAP **(A)** and L1CAM **(B)** in HIV-negative control subjects and HIV-infected women. There were significant difference in GFAP-infected exosomes **(C)** levels between N vs. SI groups [N (58.8 ± 15.5), ANI (57.8 ± 4.4), and SI (47.8 ± 18.7)] by Kruskal–Wallis test (statistic 7.276; *p* = 0.0263) with Dunn's multiple comparisons (N vs. SI *p* = 0.029). No significant differences were detected in L1CAM-infected exosome levels **(D)** between the HAND groups by the Kruskal–Wallis test with Dunn's multiple comparisons (statistic 2.408; *p* = 0.300), [N (7.7 ± 4.9), ANI (6.5 ± 4.1), SI (6.3 ± 5.5)]. Spearman's correlations were used to determine associations between the percentage of ROS+ exosomes and the levels of GFAP immunoreactivity per exosome in HIV-negative control subjects **(E)** and HIV-infected patients stratified by HAND **(F–H)**.

Intrigued by the cells of origin of these exosomes isolated from plasma, we measured the levels of GFAP and L1CAM in the purified exosome samples. GFAP is a marker of astrocytes and L1CAM is a marker of neurons. Both GFAP and L1CAM-positive exosomes were significantly increased in the plasma of HIV-infected women when compared with controls (*p* < 0.001 and *p* = 0.015, Figures 4A,B, respectively). Among the HIV-infected group, GFAP-infected exosomes are significantly higher in those with normal cognitive infection when compared with those with cognitive impairment (SI, Figure 4C; *p* = 0.029). No differences were observed in the L1CAM levels between the HAND groups (Figure 4D). No correlation was observed between exosomal GFAP levels and ROS-positive exosomes in controls or HIV-infected women with normal cognition (Figures 4E,F; *p* = 0.107 and *p* = 0.118, respectively), or asymptomatic impaired (Figure 4G; *p* = 0.115). However, a positive correlation was observed between exosomal GFAP levels and ROS-positive exosomes in HIV-infected women with symptomatic impairment (Figure 4H; *p* = 0.034).

### sIR in the Urine and eGFR Levels of HIV-Infected Women

**Figure 5:**
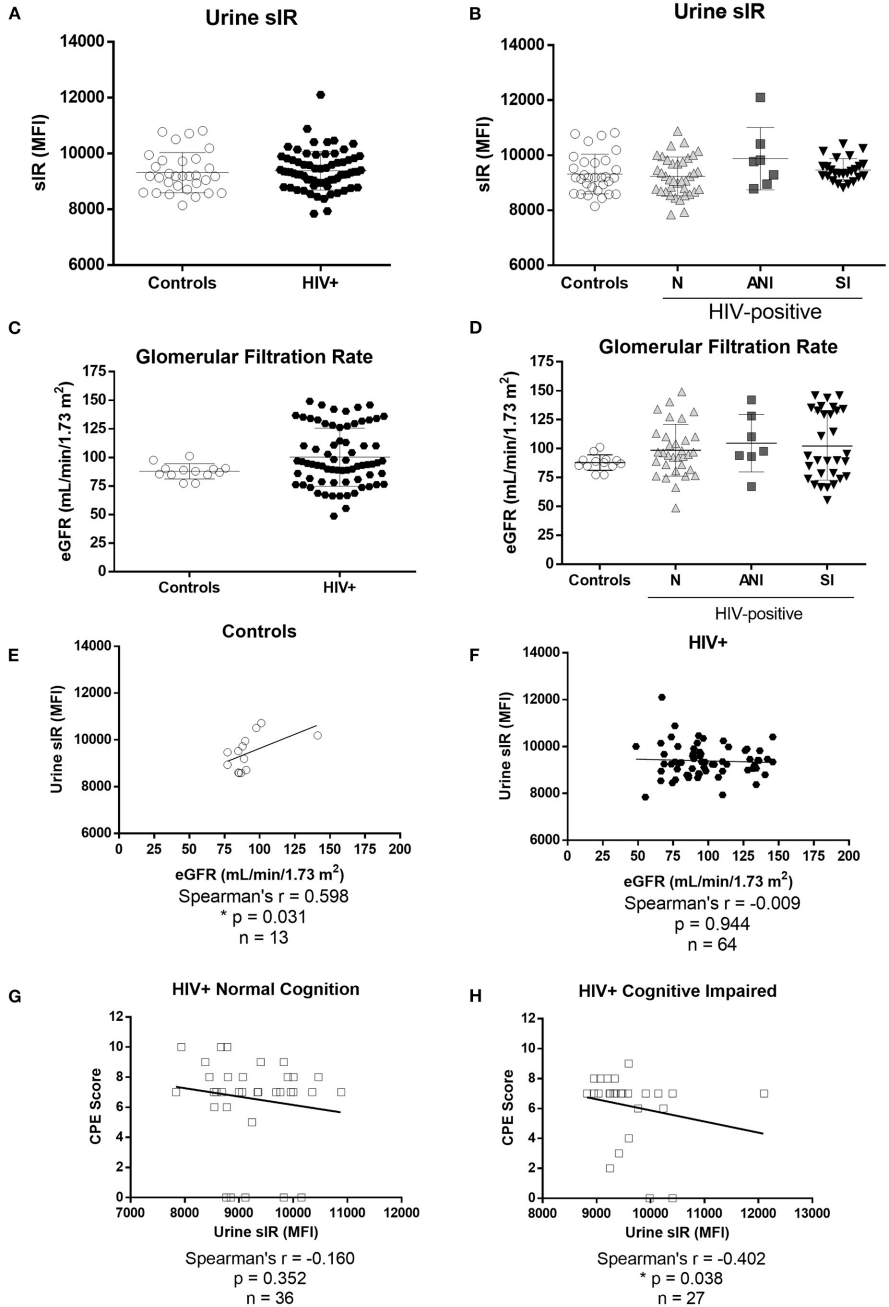
Levels of soluble insulin receptor in urine and estimated glomerular filtration rate of HIV-negative control and HIV-infected women. sIR was measured in the urine of HIV-negative (controls *n* = 31) and HIV-infected (*n* = 76) by flow cytometry **(A)**. HIV-infected women were stratified by HAND status as normal cognition (N), asymptomatic cognitive-impaired (ANI), and symptomatic impaired (SI; MND and HAD) **(B)**. eGFR (ml/min/1.73 m^2^) was measured in HIV-negative controls (*n* = 13) and HIV-infected women (*n* = 71) grouped **(C)** and stratified by cognitive function **(D)**. Flow cytometry results were graphed as mean fluorescence intensity (MFI). Graphs show mean and standard deviation [Controls (9,316 ± 719.8), N (9,230 ± 703.8), ANI (9,876 ± 1131), and SI (9,465 ± 412.2). Spearman's correlations between levels of sIR (MFI) and eGFR were conducted in urine samples from controls **(E)** and HIV-infected women **(F)**. Spearman's correlations between levels of urine sIR and CPE score were conducted in samples from HIV-infected women without and with cognitive impairment **(G,H)**.

sIR levels were successfully quantified in the urine of HIV-infected women and controls. There were no differences in urine sIR levels between controls and HIV-infected women (Figure 5A) or when the samples were stratified by HAND (Figure 5B). The estimated glomerular filtration rate (eGFR), an indicator of kidney function, was similar in controls and HIV-infected women (*p* = 0.115, Figures 5C,D). No differences in urine sIR were observed among HIV-infected women stratified by protease inhibitors use in their cART regimen (*p* = 0.46, data not shown). A positive correlation between sIR in urine and eGFR was observed in controls (Figure 5E; *p* = 0.031). However, this correlation is not present in HIV-infected women (Figure 5F; *p* = 0.944). Urine sIR levels negatively correlate with the ART CPE score in cognitively impaired HIV-infected women (Figure 5H; *p* = 0.038) but not in normal cognitive HIV-infected women (Figure 5G).

### Correlation Between Urine sIR, Plasma sIR, and Exosomal sIR

**Figure 6:**
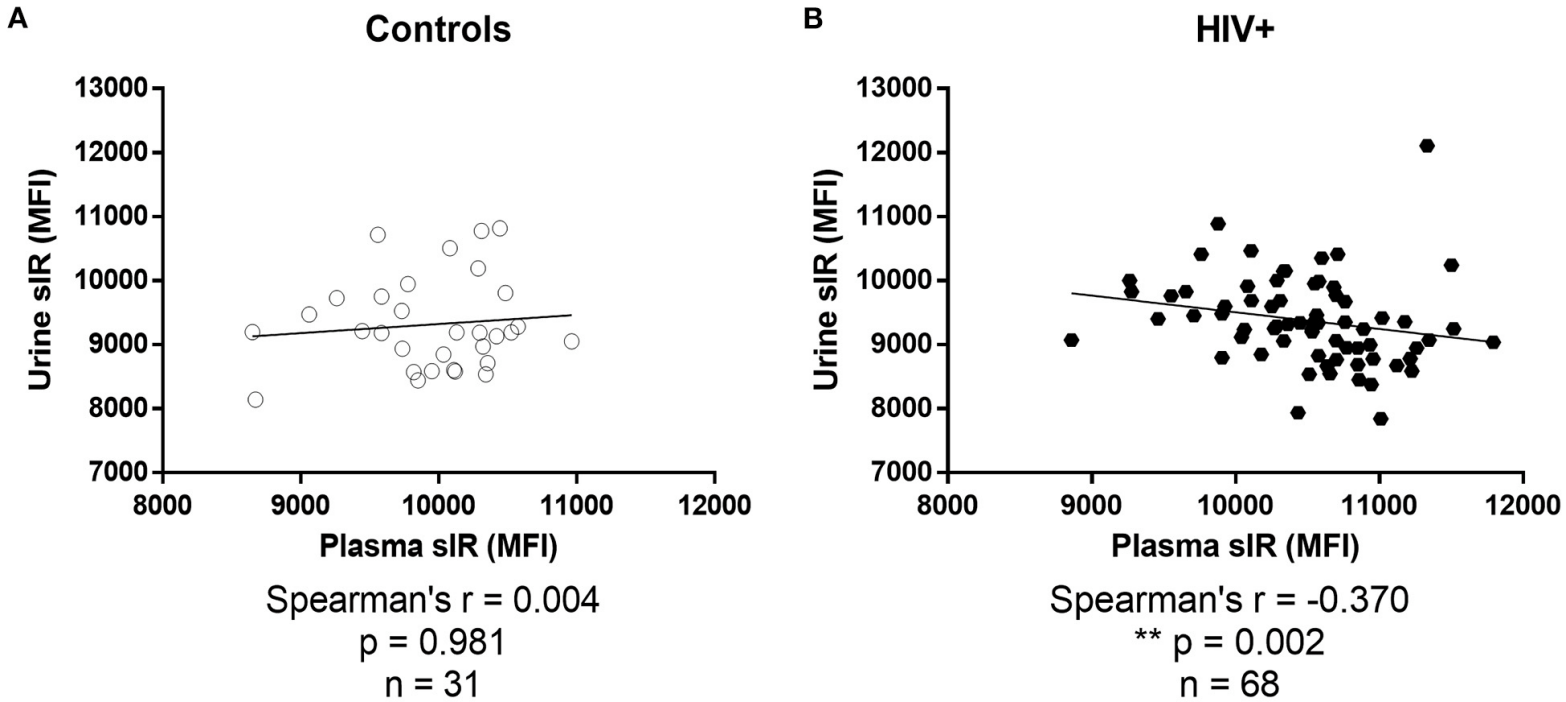
Association between soluble insulin receptor levels in urine and plasma. Associations between sIR levels in plasma and urine were determined by Spearman's correlations tests in controls **(A)** and HIV-infected women **(B)**.

No correlation was observed between urine and plasma sIR in controls (*p* = 0.981; Figure 6A). On the contrary, these parameters had a significant negative correlation in HIV-infected women (*p* = 0.002; Figure 6B).

### HCV Coinfection Increased Urine sIR but Not Plasma sIR Levels

A secondary analysis evaluating HIV/HCV coinfected patients was performed to determine the differences in sIR levels. The plasma levels were similar in HIV-infected and HIV/HCV coinfected women (*p* = 0.419; Supplementary Figure 1A). Urine levels were significantly higher in HIV/HCV coinfection (Supplementary Figure 1B, *p* = 0.020). sIR, HIV-1 Tat, ROS, and GFAP and L1CAM-positive exosomes were similar in both groups (Supplementary Figures 1C–G). There were no differences when stratified by the HAND groups (data not shown). CD163 was measured as a marker of macrophage activity and inflammation in the plasma of HIV-infected women only. Plasma CD163 did not differ between HIV-infected women stratified by cognitive impairment (*p* = 0.051; data not shown). However, HIV/HCV coinfected women had higher CD163 levels in plasma (*p* = 0.002) when compared to HIV-infected women negative for HCV (Supplementary Figure 1H). HIV/HCV coinfected patients performed similarly in neuropsychological tests (Supplementary Figure 2).

### HIV-Infected Women With Positive Toxicology Test Results Did Not Alter sIR Findings

Table 1 reports a borderline significantly higher percentage of HIV-infected women having infected toxicology results. The drugs reported as being used were cocaine (*n* = 7), marijuana (*n* = 7), or a combination of the two (*n* = 3) [3 participants did not provide a report of the drugs used].

When we compared plasma sIR levels across groups defined by HIV-infected and infected toxicology status, the levels were significantly increased in HIV-infected women without infected toxicology results [median (IQR): 10,556 (10,040, 10,889), *p* < 0.05] and with toxicology infected results [10,442 (10,085, 10,721), *p* < 0.05] compared to controls (all of whom had negative toxicology results). Differences were not seen with urine sIR between these three groups, however, similar elevated levels of ROS per exosome were observed (*p* < 0.05).

Given that no controls had infected the toxicology results, we did not include an interaction term between toxicology and HAND status. While our sample size was not sufficient to perform a non-parametric regression of plasma sIR as a function of HAND status and toxicology, we did use a log transformation of plasma sIR and conduct a linear regression with these main effects and an interaction term. Controlling for the influence of infected toxicology results, log plasma sIR was significantly elevated in HIV-infected women with symptomatic cognitive impairment.

## Discussion

PWH using cART suffer metabolic alterations that could contribute to the development of HAND (1, 2, 36). As previously observed, plasma sIR levels were significantly increased in HIV-infected women when compared with controls, especially those with symptomatic cognitive impairment (Figure 1). HIV-infected women with cognitive impairment have higher levels of sIR in plasma and CSF when compared to HIV-negative women, as well as higher insulin binding to sIR and lower levels of IRS-1 tyrosine phosphorylation in plasma (13, 14). It is not clear how sIR is released from the cells to the blood. One of the possible mechanisms of secretion is exosomes.

There are extensive data on viral proteins contained in exosomes derived from HIV-infected cells and secreted to the plasma (18, 37–41). These exosomes are potentially involved in indirect exosome-mediated neurotoxicity associated with HIV-1 infection (42, 43). Cell-derived receptors can also be secreted to the extracellular compartment *via* exosome release (44, 45) and their generation is observed during HIV infection (13–15, 46–48). Most of these receptors are dysfunctional and lack a transmembrane domain region (44, 45, 49). Our results show that sIR, ROS, and HIV-1 Tat are secreted from cells *via* exosomes in HIV-infected women and that HIV infection significantly increases the number of sIR, ROS, and HIV-1 Tat-containing exosomes in the blood of these patients. Moreover, exosomal sIR levels positively correlate with exosomal ROS and HIV-1 Tat levels in HIV-infected patients but not in controls (Figures 2F,G). Exosome levels of sIR, ROS, and HIV-1 Tat are significantly increased in HIV-infected women mainly those with symptomatic cognitive impairment. These findings suggest that metabolic derangements, oxidative stress, and HIV neurotoxins contribute to the presence and severity of HAND. The fact that exosomal sIR, ROS, and HIV-1 Tat levels did not correlate with total sIR levels in plasma, suggests that the exosomal fraction reflects changes that are not observed when analyzing the whole plasma, and may contain different biomarkers associated with HIV infection worth exploring in future studies.

An interesting finding worth discussing is the negative correlation between the percentage of exosomes containing HIV-1 Tat and the nadir CD4 T-cell count, which suggests that T-cell deficiency may predispose other cells to the secretion of HIV-1 Tat in exosomes, which may eventually lead to neuronal injury. Exosomal HIV-1 Tat may also represent a marker of HIV-associated immune dysfunction.

Our study also revealed that these exosomes have astrocyte and neuron markers on their surface, suggesting there is communication between the CNS and periphery, and that exosomes derived from astrocytes and neurons may also serve as blood biomarkers of HIV infection since they were significantly increased in exosomes from HIV-infected women. Astrocyte-derived exosomes significantly correlated with ROS-infected exosomes, pointing to an association between astrocyte activation and oxidative stress (Figure 4H). The fact that approximately 50% of the exosomes isolated from plasma are positive for astrocyte markers in the HIV-infected group confirms that HIV-1 infection activates astrocyte activity. The high levels of GFAP-positive exosomes in the plasma of PWH suggest astrogliosis and/or immune processes ongoing in the CNS despite cART treatment. It has been recently reported that human astrocytes are productively infected *in vivo* and support the spread of HIV-1 from the brain to the periphery, in a chimeric mouse model of HIV-1, which continued even after cART injection (50). Together, these results suggest that astrocytes play a significant role in neuroHIV despite cART treatment. Brain-derived exosomes have a biomarker potential for all neurodegenerative diseases and may serve as drug delivery vehicles to the CNS as well (51–59). It has been reported that neuronal-derived extracellular vesicles are present in CSF and serum of HIV-1 transgenic rats (60). In PWH, the content cargo of neuronal-derived exosomes is altered, with higher proteins associated with neurotoxicity and neurocognitive impairment, such as neurofilament light chain (NF-L) and amyloid-beta (61). Therefore, we need mechanistic studies to determine the sources, proportions, and cargo of plasma exosomes, and what is their role in HIV disease progression and/or cognitive decline.

The detection and quantification of the sIR ectodomain in urine have been previously evaluated for the early identification of patients at risk of developing diabetes mellitus (28). We were able to measure the sIR full-length levels in the urine of HIV-infected women and controls. The urine sIR levels were similar in HIV-infected women when compared to controls (Figure 5A). A significant positive correlation was observed between sIR in urine and eGFR only in controls (Figure 5E), suggesting that in control patients with normal kidney function, sIR is excreted in the urine.

Kidney function may be affected by age, hypertension, diabetes, medications including ARTs, and infections such as HIV and HCV (62). It is known that HIV can infect and replicate within the renal epithelial cells and dysregulate the epithelial cell function (63). Renal diseases secondary to HIV infection are considered a glomerular-dominant diseases and may present as HIV-associated nephropathy, acute renal failure, and chronic renal failure (63). Risk factors for kidney disease in PWH are older age, black race, higher viral loads, low CD4 cell counts, chronic use of ART, coinfection with HCV, and comorbidities such as diabetes, hypertension, and increased life expectancy—all may be contributing to the renal injury and dysfunction (62, 64–66). However, the interaction between HIV infection and the renal cell is unclarified (62). The kidney has a role in glucose regulation [reviewed in (67)]. Renal IRs are mostly in the proximal and distal convoluted tubules and insulin binding occurs in the glomeruli, renal cortex, and medulla. The kidney regulates the overall glucose homeostasis by the uptake and utilization of glucose and glucogenesis. The kidney function in our cohort was determined by the eGFR, creatine, and BUN levels, and the presence of proteinuria or albuminuria in the urinalysis, all found to be normal, and no significant differences were observed between the HIV-infected and control women suggesting that the renal filtration function is adequate. Also, most of the HIV-infected women were virally suppressed and the CD4 cell amount was higher than 200 cells/mm^3^, suggesting a low possibility of having proteinuria and renal failure as determined by Szczech et al. (68). Therefore, our finding of urine sIR may not be affected by the HIV infection or ART, although mild dysfunction cannot be completely discarded since specific biomarkers for renal injury were not tested (69, 70). As we know, high sIR levels in the plasma bind significant percentages of free plasma insulin and are associated with HAND status (14). Thus, the removal of the receptor from the circulation and subsequent excretion from the body may be beneficial for cognitive function in PWH. It is known that other peptides are removed from the circulation by glomerular filtration and account for a significant percentage of overall systemic removal from the plasma (71). On the other hand, glomerular hyperfiltration has been associated with insulin resistance in other study cohorts (72). We did not find differences in eGFR between HIV-infected and control women, nor among HAND stratified groups. However, we did find that in the HIV-infected women, the urine sIR levels did not correlate with the eGFR as it did in the control group. We also observed a negative correlation between plasma sIR and urine sIR in HIV-infected women, suggesting that at higher plasma sIR levels, there is a decrease urine secretion of sIR, an observation not present in the control group. The mechanism for removal of the insulin receptor isoforms from blood is still poorly understood. In PWH, possible explanations may include direct or combined effect of HIV infection and cART on kidney function, kidney insulin resistance, or that the IR may be trapped at other organs or tissues, therefore, longitudinal studies may be useful.

We are still not clear on the mechanism by which sIR is generated and then secreted in exosomes to the plasma of PWH and excreted in the urine. However, our findings suggest that HIV-1 infection may trigger the release of sIR to plasma, urine, and exosomes, subtly affecting neurocognitive functions in PWH. Exosome-mediated release of both sIR and HIV-1 Tat may uncover novel mechanisms to explain the pathogenic processes associated with insulin resistance and cognitive impairment in the PWH. The identification of these molecular mechanisms could assist in finding novel targets and pathways that may lead to early diagnosis, identifying risk factors, evaluating treatment effects in clinical trials, and understanding the mechanisms leading to HAND.

## Data Availability Statement

The raw data supporting the conclusions of this article will be made available by the authors, without undue reservation.

## Ethics Statement

The studies involving human participants were reviewed and approved by Oficina Para la Protección de Participantes Humanos en Investigación (IRB/OPPHI) University of Puerto Rico, Medical Sciences Campus. The patients/participants provided their written informed consent to participate in this study.

## Author Contributions

VW and YG were responsible for the conception and design of the study. VW, YC-R, YG, MM, RR, BD, RR-B, and ER contributed to the data acquisition. VW, YC-R, RS, and YG were involved in the analysis and interpretation of data. VW, YC-R, and YG drafted the first version of the article. All authors critically revised the manuscript for important intellectual content. All authors gave final approval of the version to be submitted.

## Funding

This work was supported by R01NS099036, R21MH095524, UPR-MSC Center for Collaborative Research in Health Disparities U54MD007600, NYU COMRADE Program R25NS094093, K22NS118975, and The Hispanic Alliance for Clinical and Translational Research (Alliance) U54GM133807.

## Conflict of Interest

The authors declare that the research was conducted in the absence of any commercial or financial relationships that could be construed as a potential conflict of interest.

## Publisher's Note

All claims expressed in this article are solely those of the authors and do not necessarily represent those of their affiliated organizations, or those of the publisher, the editors and the reviewers. Any product that may be evaluated in this article, or claim that may be made by its manufacturer, is not guaranteed or endorsed by the publisher.

## Supplementary Material

Supplementary Figure 1 Comparison of sIR levels measured in study participants segregated as HIV-infected and HIV/HCV coinfected women. Measures from HIV-infected women samples were divided by hepatitis C (HCV) coinfection to compare the levels of sIR in plasma **(A)**, sIR in urine **(B)**, sIR in exosomes **(C)**, HIV-1 Tat in exosomes **(D)**, ROS in exosomes **(E)**, percentage of GFAP+ exosomes **(F)**, percentage of L1CAM+ exosomes **(G)**, and CD163 in plasma **(H)**. Analyses were conducted using the Mann–Whitney test.

Supplementary Figure 2 Global and individual neuropsychological tests z-scores for study participants segregated as HIV-infected and HIV/HCV coinfected women. Z-scores from HIV-infected women were divided by hepatitis C (HCV) coinfection to compare the cognitive function **(A)** and the eight cognitive domains tested individually **(B–I)**, using an unpaired *t-*test.
