# Supplementary figures and images for: Soluble Insulin Receptor Levels in Plasma, Exosomes, and Urine and Its Association With HIV-Associated Neurocognitive Disorders
Source: Front Neurol. 2022 Jun 2;13:809956. doi: 10.3389/fneur.2022.809956 (PMC9202317; doi:10.3389/fneur.2022.809956)

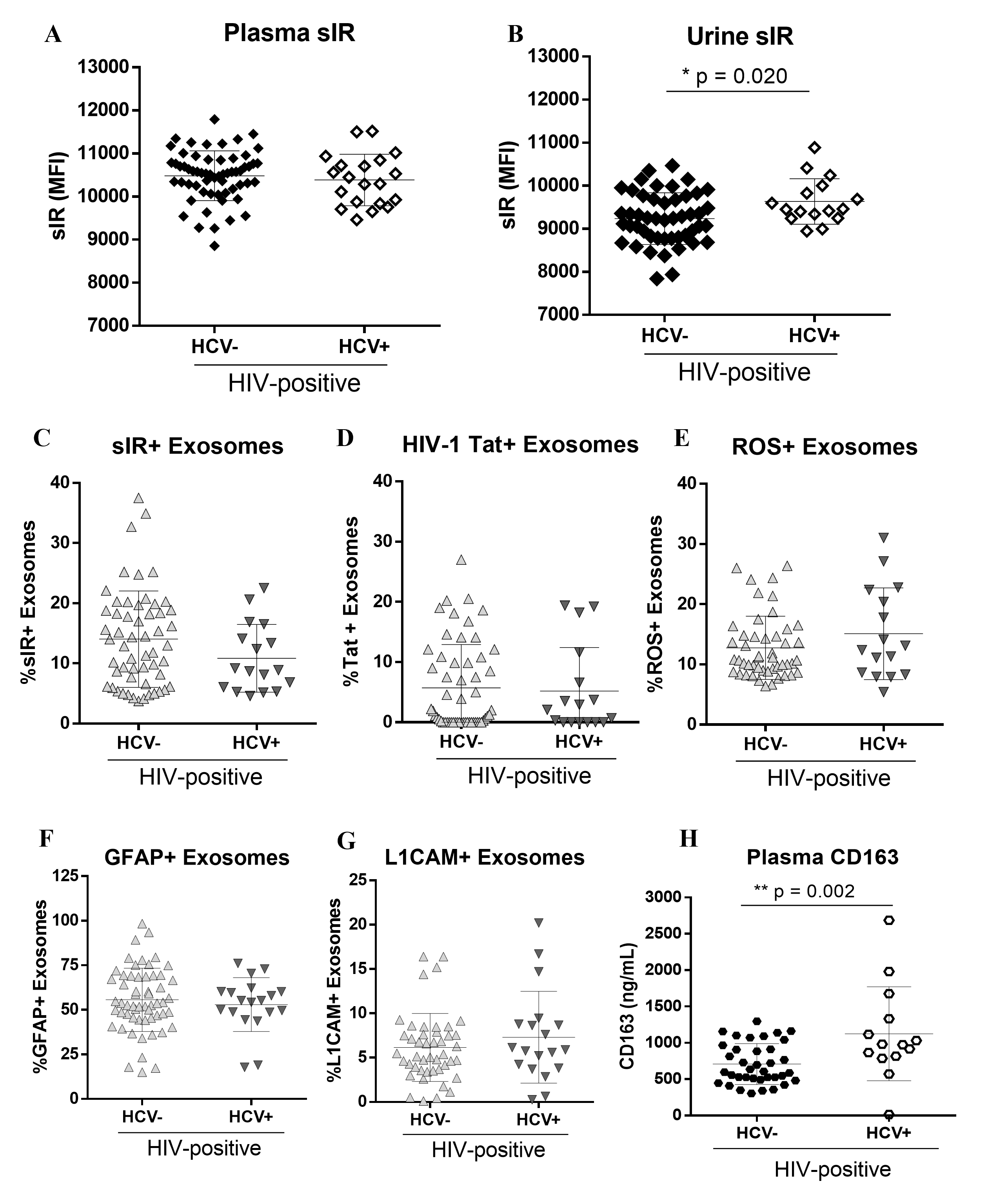

Supplement: Supplementary Figure 1 — Comparison of sIR levels measured in study participants segregated as HIV-infected and HIV/HCV coinfected women. Measures from HIV-infected women samples were divided by hepatitis C (HCV) coinfection to compare the levels of sIR in plasma (A), sIR in urine (B), sIR in exosomes (C), HIV-1 Tat in exosomes (D), ROS in exosomes (E), percentage of GFAP+ exosomes (F), percentage of L1CAM+ exosomes (G), and CD163 in plasma (H). Analyses were conducted using the Mann–Whitney test. [file Image_1.TIF]

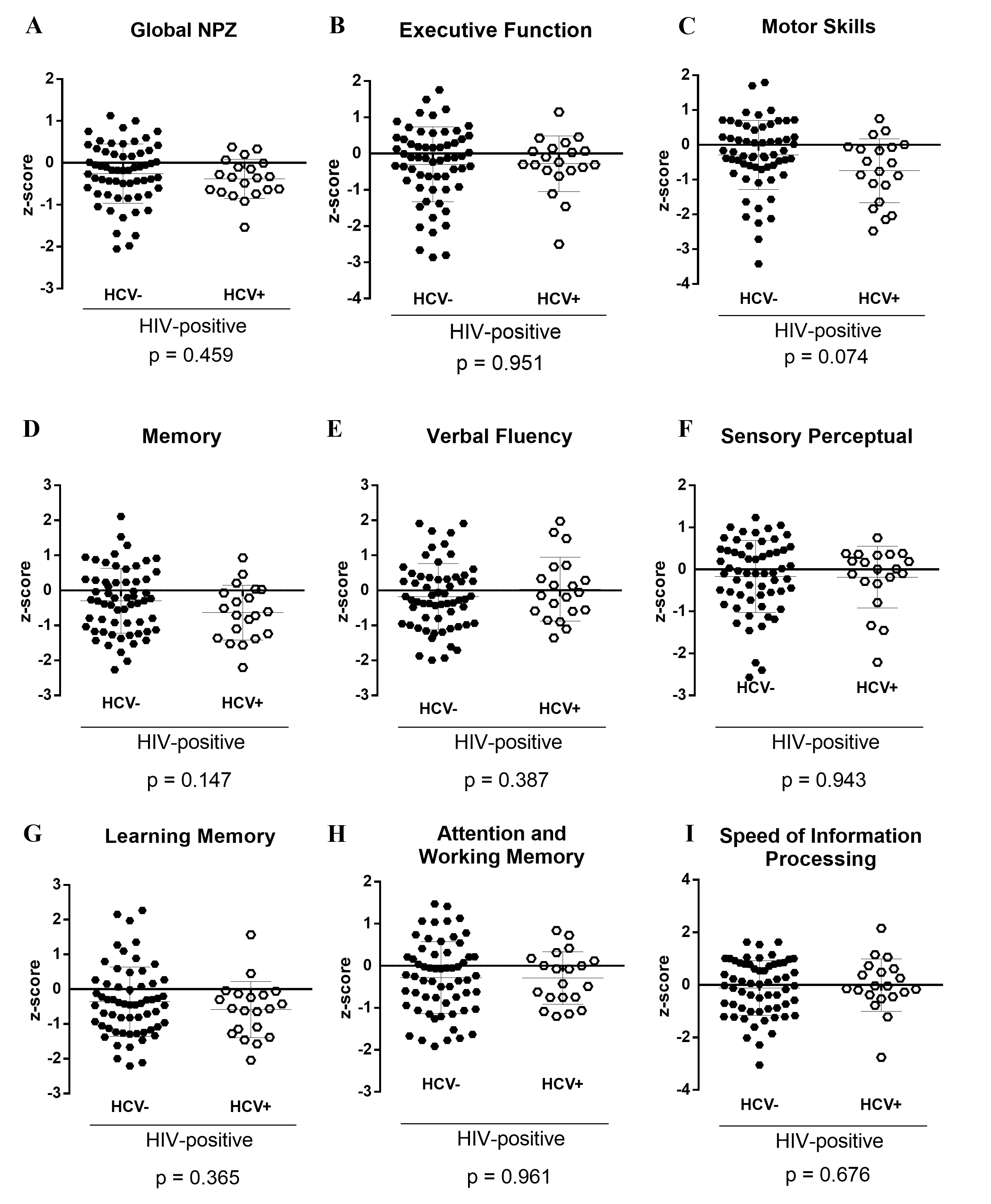

Supplement: Supplementary Figure 2 — Global and individual neuropsychological tests z-scores for study participants segregated as HIV-infected and HIV/HCV coinfected women. Z-scores from HIV-infected women were divided by hepatitis C (HCV) coinfection to compare the cognitive function (A) and the eight cognitive domains tested individually (B–I), using an unpaired t-test. [file Image_2.TIF]
